# The Effect of Therapeutic Doses of Culinary Spices in Metabolic Syndrome: A Randomized Controlled Trial

**DOI:** 10.3390/nu16111685

**Published:** 2024-05-29

**Authors:** Ayesha S. Al Dhaheri, Dana Hasan Alkhatib, Jack Feehan, Leila Cheikh Ismail, Vasso Apostolopoulos, Lily Stojanovska

**Affiliations:** 1 Department of Nutrition & Health Sciences, College of Medicine and Health Sciences, United Arab Emirates University, Al Ain P.O. Box 15551, United Arab Emirates; danakhatib@uaeu.ac.ae (D.H.A.); lily.stojanovska@vu.edu.au (L.S.); 2 Institute for Health and Sport, Victoria University, Melbourne, VIC 8001, Australia; jack.feehan@vu.edu.au (J.F.); vasso.apostolopoulos@vu.edu.au (V.A.); 3 Department of Clinical Nutrition and Dietetics, College of Health Sciences, University of Sharjah, Sharjah 27272, United Arab Emirates; lcheikhismail@sharjah.ac.ae; 4 Nuffield Department of Women’s & Reproductive Health, University of Oxford, Oxford OX1 2JD, UK; 5 Immunology Program, Australian Institute of Musculoskeletal Science (AIMSS), Melbourne, VIC 3021, Australia

**Keywords:** edible spices, ginger, cinnamon, black seed, metabolic syndrome, dyslipidemia, hyperglycemia, hypertension

## Abstract

Non-communicable diseases (NCDs) place a significant burden on global health and the healthcare systems which support it. Metabolic syndrome is a major risk factor for a large number of NCDs; however, treatments remain limited. Previous research has shown the protective benefits of edible dietary spices on key components of metabolic syndrome. Therefore we performed a 12-week double-blind, placebo-controlled, randomized, clinical trial to evaluate the effect of ginger (*Zingiber officinale*), cinnamon (*Cinnamomum*), and black seed (*Nigella sativa*) consumption on blood glucose, lipid profiles, and body composition in 120 participants with, or at risk of, metabolic syndrome. Each participant consumed 3 g/day of powder (spice or placebo). Data related to different parameters were collected from participants at the baseline, midpoint, and endpoint of the intervention. Over the 12-week interventions, there was an improvement in a number of biochemical indices of metabolic syndrome, including fasting blood glucose, HbA1c, LCL, and total cholesterol associated with supplementation with the spices when compared to a placebo. This study provides evidence to support the adjunct use of supplementation for those at risk of metabolic syndrome and its sequelae.

## 1. Introduction

According to the World Health Organization (WHO), the number of deaths caused by non-communicable diseases (NCDs) is increasing worldwide. In 2015, 70% of deaths globally were caused by NCDs, and most of these resulted from complications of four main NCDs: cardiovascular diseases (CVDs), cancers, diabetes, and chronic lung diseases [1]. Of these four, the most frequent cause of death is CVD. Of 40 million NCD deaths annually, 17.7 million (45%) are attributed to CVD [1]. Obesity, type 2 diabetes, hypertension, and high blood lipid levels are the main causes of CVD [2,3,4].

Metabolic syndrome (MetS) is a combination of medical illnesses that can include high fasting blood glucose (FBG) levels, elevated blood pressure, central obesity, high blood triglyceride (TG) levels, insulin resistance, diabetes, elevated blood low density lipoprotein (LDL) levels, and reduced blood high density lipoprotein (HDL) levels [5]. The characteristics of a MetS diagnosis are controversial, although all definitions agreed on three common characteristics as follows: reduced HDL, elevated blood pressure, and insulin resistance [5,6,7]. MetS management aims to reduce the risk of clinical factors that could lead to CVD. Changing a MetS patient’s lifestyle could help to treat symptoms and improve the quality of life for people at risk of CVD [8,9].

Dietary factors include improving the nutritional components in an individual’s diet. Reducing the fat content of the diet and managing the carbohydrate content could improve the regulation of insulin sensitivity, blood glucose, and blood lipid levels [10,11]. One method used to manage blood parameters that could affect the risk factors leading to MetS is herbal therapy. Herbal therapy is broadly used in many countries as a treatment or as a preventive measure to manage MetS risk factors, including blood glucose, blood pressure, and blood lipid levels [12,13,14,15,16]. Ginger (*Zingiber officinale*), cinnamon (*Cinnamomum*), and black seed (*Nigella sativa*) are annual plants that have been traditionally used internationally, particularly on the Indian subcontinent, Europe, and Arabian countries such as the UAE [17]. These spices were used for food preparation and medicinal purposes, often as a cure for many diseases and conditions, including diabetes, asthma, hypertension, inflammation, cough, bronchitis, headache, eczema, fever, dizziness, and influenza. Additionally, black seed is used as a diuretic, lactagogue, and vermifuge, while ginger and cinnamon are used as anti-tumor agents, and cinnamon is also used to decrease muscle soreness in athletes. These spices are also used in food as aromatic spices, carminatives, and condiments [17,18,19,20,21,22,23]. The primary objective of this study is to evaluate the effects of ginger (*Zingiber officinale*), cinnamon (*Cinnamomum*), and black seed (*Nigella sativa*) powders on FBG, hemoglobin (Hb) levels, glycated hemoglobin (HbA1c) levels, blood pressure, the blood lipid profile, and waist circumference (WC) among participants at risk for CVD.

## 2. Materials and Methods

### 2.1. Ethical Approval

This study was carried out according to the Declaration of Helsinki guidelines. The United Arab Emirates University Scientific Research Ethics Committee approved all procedures concerning human subjects (UAEU, Protocol Number: 15/22; Reference Number: DT/bb/15-22; approval date: 10 May 2015). All participants in the study provided voluntary informed consent to participate and were asked to sign a written informed consent to participate in the study. The study was registered in ClinicalTrials.gov (NCT06423248).

### 2.2. Study Population

This study is a controlled, randomized, single-blind, parallel-design study. It was conducted during the academic year 2015/2016 at United Arab Emirates University (UAEU) in Al Ain, United Arab Emirates. The study population includes adults aged between 18 and 50 years, who are at risk of metabolic syndrome or have metabolic syndrome. Participants were recruited through face-to-face interviews, printed advertisements, and via email to voluntarily participate in the study.

### 2.3. Inclusion and Exclusion Criteria

Participants were considered eligible to take part in the study after meeting the following inclusion criteria: age (18–50 years), and if they had 2 or more of the following MetS diagnostic criteria: high waist circumference (>80 cm for female and >94 cm for males), elevated blood pressure (equal to or greater than 130/85 mm Hg or use of medication for hypertension), high fasting blood glucose (equal to or greater than 100 mg/dL [5.6 mmol/L] or use of medication for hyperglycemia), lowered HDL cholesterol (less than 40 mg/dL [1.03 mmol/L] for men and less than 50 mg/dL [1.29 mmol/L] for women), and increased triglycerides (equal to or greater than 150 mg/dL [1.7 mmol/L]). Participants who had at least three out of five risk factors or had two risk factors and one borderline were included in the study.

Participants were excluded if they did not meet the following inclusion criteria: older than 50 years old or younger than 17 years old and did not meet the metabolic syndrome diagnostic criteria. Additionally, participants were excluded if they were allergic to spices, current smokers, pregnant women, lactating women, or currently taking medication, and if they refused to provide a blood sample. Also, participants with any acute illnesses or any chronic diseases such as kidney, liver, cardiovascular, and gastrointestinal diseases were excluded from the study. During the study period, participants were asked to bring the remaining spice powder to each visit. The amount left was calculated to make sure that the participants had consumed 80% of the total amount or more. Those who consumed less than 80% of the treatment powder were excluded from the study.

### 2.4. Screening Assessments

**Figure 1:**
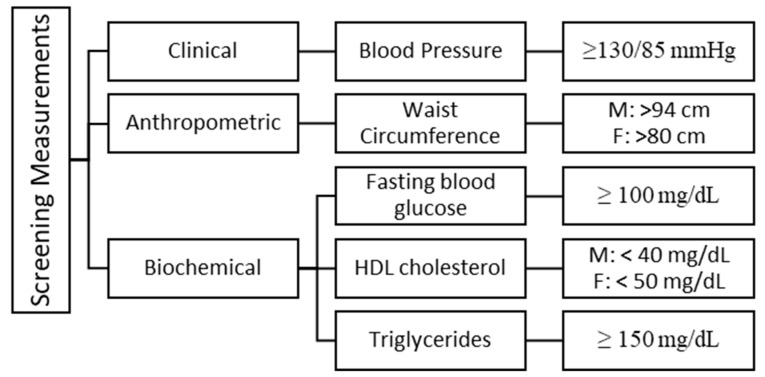
Screening criteria for metabolic syndrome in the study. M: Male, F: Female.

To ensure that the selected study population are eligible to participate in the study and had two or three out of five MetS risk factors, screening measurements were conducted for the MetS diagnostic criteria, including blood pressure (BP), waist circumference (WC), fasting blood glucose (FBG), high density lipoprotein (HDL), and triglycerides (TG) (Figure 1).

In addition, participants were asked to complete a health screening questionnaire which contains questions about medical conditions and medications that might influence glucose regulations, appetite, and energy expenditure.

### 2.5. Intervention

A 12-week clinical randomized intervention was used to compare three treatments (ginger, cinnamon, and black seed) with those from the control group (placebo). Participants were divided randomly into four groups and asked to consume three grams per day, on separate occasions, of the allocated spice or corn starch powder for 12 weeks. Each participant was asked to consume one gram in the morning, one gram in the middle of the day, and one gram at the end of the day with hot water. Data was collected from participants at three points as follows: the baseline, midpoint (six weeks), and endpoint (twelve weeks) of the study period.

The spices were purchased from a local spice shop in Al Ain city, with the whole amount purchased at once to minimize any error. The spices were freshly ground into a powder for the intervention study. In a clean and sanitized area, the spice powder and starch powder were weighed into one-gram portions and were packed in small zip lock plastic bags for the convenience of the participant’s usage.

Once participants met all of the inclusion criteria, they were asked to maintain the same dietary habits and physical activity levels for 12 weeks. They were asked to record their dietary intake using a three-day food record, and to report their physical activity level with the International Physical Activity Questionnaire (IPAQ). The mentioned records were submitted by the participants at each visit to ensure that there were no changes in dietary and physical activity habits introduced throughout the study period.

### 2.6. Anthropometric Measurements

All anthropometric measures in this research were taken in a thirty-minute visit at the baseline, midpoint, and endpoint of the study period. These measures were taken by a researcher who had received training on anthropometric measurements. Participants were asked to attend each visit after fasting for 10–12 h. Measurements were taken in triplicates, and the average of the readings was calculated and used in the study. Anthropometric measures included height, weight, body composition, and waist circumference. Considering the limits of local culture, participants took the anthropometric tests wearing minimal clothes and with no shoes on. Devices used in the study were calibrated daily to ensure accuracy. At each visit, participants were asked to rest for 15 min to adjust for climate and physical activity changes.

Height was recorded to the nearest centimeter using a stadiometer (Seca Stadiometer, Seca Ltd., Birmingham, UK), with participants standing straight and without wearing shoes, heavy outer garments, hair covers, or hair accessories. Each participant stood erect with their head in the Frankfurt plane with ears and cheekbones at the same level, and the arms at the sides, feet positioned close together, knees straight, and with their heels, buttocks, and shoulder blades touching the vertical surface of the stadiometer as recommended by the WHO STEPS protocol [24]. Body weight and body composition were recorded to the nearest 0.1 kg using the InBody 270 (InBody, Seoul, Republic of Korea) and the Hologic Discovery Dual X-ray Absorptiometry (DXA) system, with participants wearing light clothing (due to cultural limits) and with no shoes or socks on.

Participants were asked to stand unassisted on the body composition analyzer platform (with no shoes or socks on), placing their heels on the posterior electrodes, and with the front part of the feet in contact with the anterior electrodes. Participants were asked to hold the grips with both hands and wait 15–120 s until the device completed its measurements. A certified radiologist conducted all DXA imaging, with participants being asked to remove all objects from pockets; however, false teeth, hearing aids, jewelry, and watches did not have to be removed. Women that met the inclusion criteria were asked to take a pregnancy test and to self-report that they were not pregnant. If they were pregnant, they were automatically excluded from the study for the safety of the embryo.

The BMI was calculated from weight and height using the following formula: (weight (kg)/height (m^2^)) [25]. The measurement of the waist circumference (WC) was carried out using a plastic tape and was recorded to the nearest centimeter. A stretch-resistant tape that provides a constant 100 g of tension was used to measure the WC. Participants were asked to stand up erect with the arms at the sides, feet positioned close together, and weight equally distributed on the feet. The tape was placed at the approximate midpoint between the lower margin of the last palpable rib and the top superior border of the iliac crest, or at the umbilicus level for obese participants according to The WHO STEPS protocol [24]. Three measurements were obtained, and the average of the readings were recorded.

### 2.7. Dietary Assessment

Participants were asked to sustain their dietary habits and not to change them during the 12 weeks of study. A three-day food record was collected from each participant on each visit (baseline, midpoint, and endpoint), totaling nine days of food records for each participant. At the beginning of the study, the researcher sat with each participant and demonstrated how to fill the food record using food models, visual aids, and measurements tools.

Participants were taught how to record their three-day food record, accurately and in detail (for example, brand name, fat percentage, fortification, etc.). Food records were analyzed using Food Processor Nutrition Analysis software (ESHA). When traditional Emirati and local food were consumed, Emirati and Kuwaiti food composition tables were used.

### 2.8. Physical Activity Assessment

The International Physical Activity Questionnaire (IPAQ) was used to ensure that the participants did not change their physical activity habits during the study period. Guidelines for data processing and the analysis of the IPAQ were used to analyze the data gained [26].

### 2.9. Biochemical Assessments

Blood collection was performed after 10–12 h of fasting by a trained phlebotomist, with samples being collected into Ethylenediaminetetraacetic acid (EDTA) vacutainers. After blood collection, tubes were placed in a centrifuge (Heraeus^®^, Biofuge Primo R, Kendro, Germany) at 1500 rpm for 15 min, with serum being collected from the blood tubes and stored in 1 mL Eppendorf tubes (Eppendorf^®^, Safe-Lock micro test, 2015, Hamburg, Germany) at −80 °C.

Fasting blood glucose (FBG), triglycerides (TG), high density lipoprotein (HDL), low density lipoprotein (LDL), and total cholesterol were measured using the Cobas C111 analyzer^®^ (Roche Diagnostics Ltd., Mannheim, Germany). Serum samples for each participant from the three phases (baseline, midpoint, and endpoint) were measured together, in the same run, to minimize the chance of error. Reagents, controls, and calibrations of the Cobas C111 were used according to manufacturer guidance and training protocols.

### 2.10. Blood Pressure

BP was measured using an Omron professional blood pressure monitor (HEM 907, Tokyo, Japan). Participants were given 15 min of rest to adjust to the climate changes. The measurement was taken twice with 5 min breaks between measures, and the average measurement was recorded.

### 2.11. Statistical Analysis

Power was calculated at 80%, referring to previous articles that studied the effect of similar spices [27,28,29], and, considering Alpha = 0.05, the sample size required for the experiment was calculated using the Minitab software, at 17 per group. In addition, to account for approximately 40% attrition due to the long follow-up, the target recruitment was 30 participants for each group, with a total of 120 participants. Participants were randomized with randomization software with permuted blocks of 10. Data analysis was carried out using the IBM Statistical Package for Social Sciences SPSS^®^ software (version 23.0) (IBM Corp, Armonk, NY, USA). Descriptive statistics were computed and summarized; continuous variables were summarized using means and standard deviations (SD). The normality of the data was assessed through Shapiro–Wilk testing. Linear mixed models were used to evaluate the differences between the interventions over time. The alpha was set to 0.95, therefore *p* values less than 0.05 were considered statistically significant.

## 3. Results

### 3.1. Recruitment and Randomization

**Figure 2:**
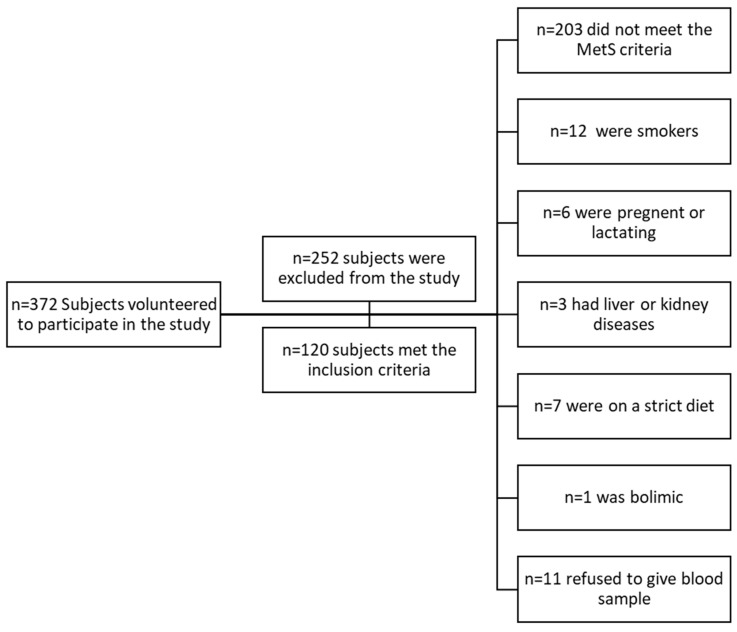
Participant screening and inclusion.

A total of 372 individuals expressed interest in the study and were initially screened for eligibility at the Nutrition Clinic of UAEU. Of these, 252 were excluded from the study for the following reasons: 203 did not meet the metabolic syndrome diagnostic criteria nor were at risk for developing metabolic syndrome, 12 were smokers, 6 were pregnant or lactating, 3 had liver or kidney diseases, 7 were on a strict diet, 1 was bulimic, 9 did not want to sign the consent form, and 11 did not consent to giving blood (Figure 2).

**Figure 3:**
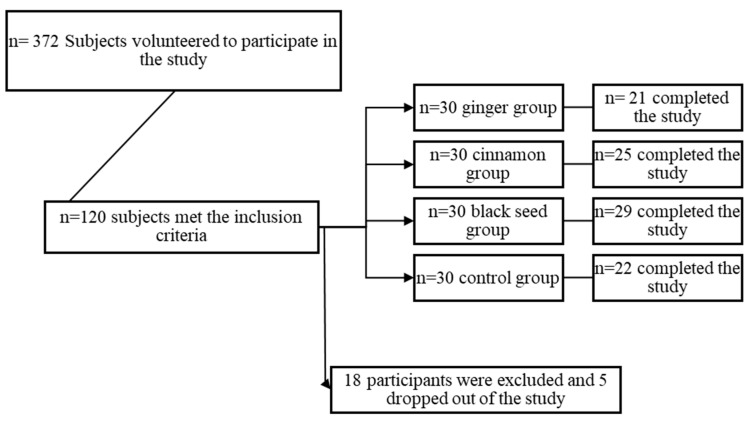
Recruitment and participant dropout.

Of the 120 participants recruited, 18 were excluded from the study due to failing to consume 80% of the allocated treatment (n = 8), major changes to their diet (n = 6), increasing their physical activity levels (n = 6), and an additional 5 dropped out from the study for personal reasons. A total of 97 participants completed the full 12 week intervention (Figure 3).

### 3.2. Population Characteristics

The clinical and demographic characteristics of the study population are presented as mean ± standard deviation in Table 1. The mean age of the study population was 26.59 ± 8.07, 27.84 ± 12.04, 26.10 ± 9.57, and 28.82 ± 11.70 years old for the black seed, cinnamon, ginger, and placebo groups, respectively, ranging from 19 to 49 years old. The mean body mass index (BMI) of the participants was 34.78 ± 9.28, 33.53 ± 9.96, 36.07 ± 6.47, and 33.94 ± 5.84 kg/m² in the black seed, cinnamon, ginger, and placebo groups, respectively.

All the participants had two or more of the metabolic syndrome risk factors. Waist circumference (WC) was 98.61 ± 17.64, 96.56 ± 13.77, 105.46 ± 14.93, and 102.08 ± 11.92 for the black seed, cinnamon, ginger, and placebo groups, respectively. The systolic blood pressure mean values were 115.48 ± 17.72, 121.68 ± 15.65, 119.05 ± 17.75, and 122.50 ± 16.79 mmHg in the black seed, cinnamon, ginger, and placebo groups, respectively, and 76.72 ± 13.44, 81.36 ± 11.25, 83.48 ± 13.42, and 81.86 ± 9.53 mmHg for the diastolic blood pressure in the black seed, cinnamon, ginger, and placebo groups, respectively. The fasting blood glucose (FBG) mean values were 93.69 ± 8.47, 99.06 ± 42.62, 82.64 ± 15.03, and 78.26 ± 26.78 mg/dL for the black seed, cinnamon, ginger, and placebo groups, respectively. The high-density lipoprotein (HDL) mean values ranged from 34.81 ± 10.05 mg/dL for the ginger group to 41.36 ± 12.76 mg/dL for the cinnamon group, with no significant differences between the ginger, cinnamon, and black seed groups and the placebo group. The triglyceride mean values were the highest in the cinnamon group, 116.63 ± 79.37 mg/dL, and the lowest in the black seed group, 101.29 ± 33.00 mg/dL. No significant differences were found between the ginger, cinnamon, and black seed groups and the placebo group for triglycerides.

The participants demonstrated no significant differences between each treatment group and the placebo group in terms of their age, height, BMI, WC, BP, FBG, HDL, and TG. On the other hand, the weight mean values of the participants ranged from 92.74 ± 25.45 kg for the black seed group to 100.6 ± 21.12 kg for the ginger group. Significant differences were observed in the weight mean values between the placebo group and ginger group and between the placebo group and cinnamon group, as demonstrated in Table 1.

Effect of spice interventions on cardiometabolic factors.

After 12 weeks of treatment, there were statistically significant changes in the total and LDL cholesterol levels, as well as the fasting blood glucose and HbA1c. There were no other statistically significant changes in cardiometabolic outcomes over the duration of the study (all *p* ≥ 0.05). Both blackseed and ginger caused significant decreases in the total cholesterol (−13.055 mg/dL and −11.49 mg/dL, respectively, *p* = 0.031), as well as LDL cholesterol (−2.142 mg/dL and −5.314 mg/dL, respectively, *p* ≤ 0.001), while both the cinnamon and placebo groups showed no effects. All three spices caused small decreases (*p* ≤ 0.001) in fasting blood glucose, with blackseed showing the greatest effect (−7.426 mg/dL), followed by ginger (−3.404 mg/dL) and then cinnamon (−1.27 mg/dL). Conversely, cinnamon showed the greatest effect on HbA1c, reducing it by 0.596%, followed by smaller changes in ginger (−0.291%) and blackseed (−0.008%), as compared to a small increase in the placebo group (+0.04%, *p* ≤ 0.001) (Table 2).

Effects of spices on anthropometric indices

There were no statistically significant effects of the 12-week spice intervention on the anthropometric parameters (all *p* > 0.05) (Table 3). There was a borderline trend of a decrease in the waist circumference in the blackseed group (*p* = 0.051), with a mean change of −10.56 cm over the 12-week intervention. This aligned with small, but non-significant reductions in the visceral fat and body fat percentages; however, again, these were small and non-uniform.

## 4. Discussion

This study is the first study that examines the effect of ginger, cinnamon, and black seed powder on individuals at high risk of developing cardiovascular disease in the population of the United Arab Emirates. It is also one of the few studies measuring the effect of ginger, cinnamon, and black seed powder on people who are at risk of developing heart diseases and have two or three out of five metabolic syndrome risk factors.

The findings from the current study indicate that the consumption of 3 g/day of spices over 12 weeks have a varied impact on some indices of cardiometabolic health, but not on associated anthropometric factors. In particular, the blackseed and ginger interventions had the greatest effects on cholesterol and blood glucose.

Ginger has been evaluated as a hypoglycemic and cholesterol-lowering agent by several authors. Jafarnemjad and his colleagues, who published a meta-analysis, concluded that ginger supplementations (tablet, capsules, powder, or rhizomes) significantly lowered FBG and TG levels and significantly improved HDL [30]. Moreover, a randomized double-blinded placebo-controlled clinical trial experimented the effect of two grams of ginger powder on forty obese women. Their findings revealed a slight but not significant positive effect of ginger powder supplementation on serum blood glucose, a significant effect on TG levels when compared to the placebo [31].

The glycemic effect of ginger is believed to be due to its high content of gingerols, paradols, and shogaols. Additionally, 6-Paradol and 6-Shogaol are chemicals presented in ginger that give the ginger its pungent smell and taste. In 2017, Wei et al. published research stating that 6-paradol and 6-shogaol proved effective activity in stimulating glucose use by adipocytes and myotubes in a high-fat diet-fed mouse. The effects were credited to the upsurge in 50 adenosine monophosphate-activated protein kinase (AMPK) phosphorylation that are found in adipocytes [32]. In addition, Li et al. published a study in 2012. His study concluded that the activity of (S)-(8)-gingerol proved to be correlated with an incline in the surface distribution of glucose transporter type 4 (GLUT4) protein. This protein is responsible for glucose uptake on the plasma membrane, which enhances the glucose uptake and insulin sensitivity [33].

Ginger was believed to lower lipid levels due to its ability to increase pancreatic lipase and amylase [34]. Furthermore, ginger consumption inhibits lipid hydrolyze in intestines, which will result in lower lipid absorption and, therefore, a lower lipid profile [35]. Moreover, the findings of Hashimoto et al. conclude that 6-shogaol, found in ginger, increases intestinal efficient movement which could contribute to the improvement of lipid profile levels [36,37].

Fuhrman et al. stated that the ingestion of 250 mg/day of ginger extract for 10 weeks reduced the TG levels. Moreover, Fuhrman et al. reported that ginger extract lowers the total cholesterol levels and inhibits LDL oxidation, hence improving lipid profiles and lowering the risk of CVD [38,39]. In contrast, Bhandari et al. examined the effect of ginger extract on cholesterol-fed rabbits for 10 weeks; the study results showed that ginger reduced the total cholesterol and triglyceride level in the blood, while the reduction was only significant for the triglycerides [40]. Similarly, a study published by Verma et al. examined the effect of the daily consumption of four grams of ginger powder on rabbits for three months. Their work showed that ginger powder had no effect on reducing the total cholesterol, LDL, and triglyceride levels [41]. The current study findings were consistent with the studies of Jafarnemjad et al., Attari et al., Fuhrrman et al., and Bhandari et al. [30,31,38,40,41]. However, Verma et al. reported a different finding.

While our findings broadly align with previous research on ginger supplementation, there is a greater disagreement around cinnamon. In 2006, Ziegenfuss et al. concluded that the ingestion of 500 mg/day of cinnamon extract for 12 weeks led to an improvement in FBG and body composition [15]. Another study published in 2003 by Khan et al. examined the effect of cinnamon when given in three different doses for three different groups (group 1 (1 g/day), group 2 (3 g/day), and group 3 (6 g/day)) of diabetic people for 40 days. Their study showed that cinnamon powder decreased the FBG, total cholesterol, triglycerides, and LDL levels significantly [20]. On the other hand, a meta-analysis of randomized controlled trials focusing on cinnamon, conducted in 2008 by Baker et al., concluded that cinnamon use did not significantly improve HbA1c or FBG [42]. A meta-analysis for 10 randomized controlled trials that observed the effects of ingesting cinnamon at a dose of between 120 mg/day and 6 g/day for 4 months on a total of 543 diabetic patients, concluding that consuming cinnamon led to a significant reduction in the levels of FBG, total cholesterol, LDL, and triglyceride levels, and an improvement in HDL levels. However, cinnamon had no significant effect on HbA1c [43]. On the contrary, a randomized control trail conducted by Crawford et al. to examine the effect of consuming 1 g per day for 90 days on diabetics resulted in significantly lower HbA1c levels [44]. The current study findings are consistent with Ziegenfuss et al. [15], Khan et al. [20], Allen et al. [43], and Crawford et al. [44]. However, the study findings of Baker et al. [42] were not in agreement with the findings of the current study.

Cinnamaldehyde and cinnamic acid, found in cinnamon, have been suggested to play an important role in preventing CVDs. They both have the ability to produce nitric oxide and both have anti-inflammatory effects [45]. Camacho and his colleagues examined the effect of cinnamon on obese mice for five weeks with a diet containing cinnamaldehyde. Camacho et al. found that cinnamaldehyde significantly decreased body weight and enhanced glucose tolerance [46]. Similarly, Saifudin et al. stated that cinnamaldehyde inhibited protein tyrosine phosphatase-1B (PTP-1B), which helped in preventing type 2 diabetes and obesity [47]. In addition, a study published in 2016 proved that cinnamon supplementation reduced only the insulin resistance index. [48]. Furthermore, another study, which was published in 2012, suggests that cinnamon could have a positive effect on normalizing postprandial glucose responses in non-obese and obese adults, and, in turn, this will help in weight management [49]. In 2008, a study was published to examine cinnamon’s effect on insulin sensitivity in diabetic adults, which resulted in the significant improvement of FBG, lipid profile, blood pressure, and body fat percentage levels, with an elevation in lean body mass [50]. A double-blind, randomized, placebo-controlled clinical trial was conducted on forty-four diabetic patients who consumed three grams per day of cinnamon supplements for 8 weeks. The findings from the study suggested that cinnamon supplements significantly decreased the levels of fasting blood glucose, HbA1c, triglyceride, weight, BMI, and body fat mass when compared to the baseline; however, this was not the case when compared to the placebo group. Therefore, the reduction was not considered significant [51].

Cinnamon stimulates glucose uptake through regulating the expression of GLUT4 and through acting as an insulin mimetic that leads to the stimulation of the translocation of GLUT4, therefore causing a reduction in blood glucose levels [52,53]. Moreover, polyphenols, found in cinnamon, have been shown to improve insulin sensitivity. This effect could help in controlling blood glucose levels, and, therefore, better blood glucose management could be achieved [50]. Jarvill et al. concluded that cinnamon extracts activate insulin receptor kinase and inhibit the dephosphorylation of insulin receptors, thus causing the maximal phosphorylation of the insulin receptors. This phosphorylation is associated with improved insulin sensitivity, and has been linked to improved lipid profiles and glycemic responses [53]. Moreover, cinnamon has the ability to inhibit hepatic reductase activity, hence lowering lipid profile levels [54].

Additionally, chromium (Cr) and polyphenols found in cinnamon have significant effects on insulin signaling and glucose control. Cr was shown to improve all metabolic syndrome risk factor signs in human subjects. Cr decreases the cortisol concentration [55], which is important for weight management, as it increases insulin circulation and fat accumulation [56]. Therefore, consuming cinnamon supplements could reduce PBF, BFM, WC, and body weight measurements. A meta-analysis published in 2003 has reported a significant reduction in body weight caused by CR-supplement consumption [57]. Similarly, Anderson et al. reported that 500 mg of water extract supplementation with cinnamon for 6 weeks lowered the fasting blood glucose, total cholesterol, and LDL levels, and enhanced the insulin sensitivity of diabetic individuals [58]. The findings from Jarvill et al. and Anderson et al. are in agreement with the findings of the current research study [53,58]. Previous study findings agree with the findings of the current study when administrated in similar dosages and durations.

The findings of this current study concluded that ingesting 3 g/day of black seed powder for 12 weeks significantly reduced cholesterol, LDL, and glucose indices.

Earlier research concluded that black seed oil contains a major bioactive component, which is thymoquinone (TQ) at 30–48% [18]. TQ is a chemical compound that is known for its therapeutic potential. Most black seed’s positive effects on health are believed to be due to TQ. TQ inhibits the electrogenic intestinal absorption of glucose and therefore improves glucose levels [59]. Moreover, the fixed oil of black seed accompanied with TQ supplements displayed anti-eicosanoid and antioxidant activity, which can inhibit eicosanoid generation and therefore lower lipid profile levels [60].

A study by Heshmati J et al. in 2015 showed that the supplementation of 3 g/day of black seed oil changed significantly the FBG and HbA1c, total cholesterol, TG, HDL, and LDL levels in the blood in the intervention group after 12 weeks of consumption [61]. In addition, multiple researchers have reported that black seed has anti-diabetic and hypoglycemic activity, as the components of black seed decrease oxidative stress and thus preserve the pancreatic beta cell integrity [62].

Previous research supported that black seed improves the lipid profiles significantly [62,63]. Studies conducted by Heshmati J and Namazi N. in 2015 showed that 1 g/day of black seed powder for 12 weeks increased HDL levels. In addition, 2 g of black seed powder decreased total cholesterol, LDL concentrations, and TG levels [63]. Moreover, a randomized controlled trial was conducted by Ibrahim R et al. in 2014 to examine the effect of black seed consumption on menopausal women for 2 months. The study reported an improvement in the lipid profiles (decrease in total cholesterol, LDL, and TG, and an elevation in HDL levels) [64]. In addition, a double-blind randomized controlled study was carried out by Amin et al. in 2015. In this case, the intervention group received two grams/day of black seed powder for a duration of four weeks. The study proved the significant effect of black seed on lowering the total cholesterol, LDL, and TG levels. The reduction of TG levels is a result of the presence of nigellamin, which acts like a clofibrate (hypolipidemic agent) [65].

Additionally, this study concluded that there was a decline in the total cholesterol and LDL levels when consuming ginger and black seed powder. This could potentially be explained through the antioxidative action of thymoquinone in black seed and of gengerols and shogaols in ginger [66,67]. As well as this, the reduction could be due to the ability of black seed to increase the secretion of cholesterol in the bile and, hence, excretion in feces [68], therefore lowering the total cholesterol level in the blood.

In contrast, Najmi et al. tested the effect of black seed consumption on body composition, WC, lipid profile, and blood glucose. Sixty participants at risk for CVD consumed 2.5 mg of black seed oil twice daily for six weeks. Only the FBG, total cholesterol, and LDL levels were reduced significantly, while black seed oil had no significant effect on body composition, nor on WC [16]. Another study conducted by Shah et al. tested the effects of black seed on metabolic syndrome risk factors. The study reported a significant improvement in HDL, LDL, and FBG levels, while it had no significant effect on blood pressure, TG, and WC measurements [69]. Previous studies are consistent with the findings of this current study when administrated in a similar dosage and for a similar duration.

Previous studies investigated the effect of ginger, cinnamon, and black seed powder on CVD risk factors using different dosages. The dosage of these spices varied from 1 g per day to 3 g per day [63,70], while the duration varied from 6 weeks to 16 weeks [70,71]. Mahluji et al. reported no significant effect of ginger powder on the FBG, TG, HDL, and HbA1c levels when investigating the effect of 2 g/d of ginger for 12 weeks on blood glucose and lipid profiles [70]. In contrast, Arablou et al. assessed the effect of 1.6 g/day of ginger powder for 12 weeks on inflammatory markers, blood glucose, and lipid profiles to find that ginger powder decreased the FBG, HbA1c, TG, total cholesterol, and CRP levels significantly, while it had no significant effect on HDL and LDL [72]. Moreover, Mozaffari and his colleagues proved that the consumption of 3 g/day of ginger powder for 8 weeks caused a significant decrease in FBG and HbA1c levels, while no significant effect was noted for weight and BMI [73]. Similarly, cinnamon had a significant effect only on FBG levels when ingesting 3 g/day for 16 weeks, although no significant effect was reported in terms of HbA1c and lipid profiles as Mang et al. reported [54]. A study conducted by Vanschoonbeek et al. concluded that the consumption of 1.5 g/day of cinnamon powder for 6 weeks had no significant effect on FBG levels nor the lipid profile [74]. Moreover, Akilen et al. reported a significant effect of cinnamon powder when consuming 2 g/day for 12 weeks in HbA1c and blood pressure measurements, while the WC, FBG, weight, BMI, and lipid profile levels demonstrated no significant difference [75]. On the other hand, black seed powder was proven to show a significant effect on FBG, HbA1c, TG, and HDL levels when consumed in 3 g/day for 12 weeks. However, it exhibited no significant effect on total cholesterol and HDL, as reported by Hashmati et al. In contrast, Qidwai et al. examined the effect of 1 g/day of black seed powder for 6 weeks on blood glucose, lipid profile, WC, and blood pressure measurements. Qidwai et al. concluded no significant effect of black seed powder on the previously mentioned parameters [76]. Previous studies that involved less than 12 weeks or 2 g or less of the spice powders in their intervention recommended longer interventional periods and a higher dose of the spice powders to exhibit a significant effect of spice powder consumption on the lipid profile, blood glucose, WC, weight, and BMI [63,72,73,74,75].

In contrast, ginger, cinnamon, and black seed powder were proven to have a reverse health effect when consumed in large amounts. Toxicity, irritation, diarrhea, inflammation, and damage can be caused due to the misuse of these spices [77,78]. However, the safety and tolerability of ginger, cinnamon, and black seed was proven when consumed in doses of 3 g/day in previous studies [76,79,80].

Therefore, to increase the possibility of achieving significant findings in this current study and to assure safety, 3 g per day of the treatment for 12 weeks was administered.

In conclusion, previous study findings are consistent with our recent study results. This study indicates that the consumption of 3 g/d of ginger, cinnamon, and black seed powders daily for 12 weeks had a significant effect on improving WC, body composition, blood glucose, and the lipid profile. These results readily promote the use of adjunctive spice supplementation to manage individuals with, or at risk of, MetS, as they are likely to be safe, and provide broad spectrum cardiometabolic benefits to patients.

The strengths and limitations of this study should also be noted. In terms of strengths, this study was conducted on a human sample rather than animals, and thus the results reflect the effect on humans directly; the subjects were both male and female and were of different adult ages; both the dietary intake and physical activity levels were taken into consideration, with these levels being maintained throughout the study so as to show the effect of black seed powder only; and the body compositions were measured using different efficient ways (In body 720 and DEXA) in order to monitor body changes. In terms of limitations, participants were not familiar with food portion sizes, which required several explanations to make sure that they recorded the right portions in the food dairy records; some of the participants were either under- or overestimating their physical activity levels, so repeating the questions of the IPAQ with further explanations required more time too; in addition, not all participants adhered to the consumption 100%. Broadly, the cohort was also relatively young in the context of MetS, which is more common in middle age. It is possible that the interventions may be less effective in older adults, and future studies should include older populations or subgroups to evaluate this further.

## 5. Conclusions

The high dose supplementation of the evaluated spices led to improvements in several important indices in metabolic syndrome, including total cholesterol, LDL cholesterol, FBG, and HbA1c levels. These factors are central to both the development and exacerbation of many non-communicable diseases, making well-tolerated and widely available adjunctive therapies critically important. While larger studies are required to fully validate the effects, including differing doses and long-term supplementation, the data provided herein provide a strong rationale for future research.

## Figures and Tables

**Table 1:** Baseline characteristics of the groups.

Parameter	Black Seed (Mean ± SD)		Cinnamon (Mean ± SD)		Ginger (Mean ± SD)		Placebo (Mean ± SD)	
Age (years)	26.59	±8.07	27.84	±12.04	26.10	±9.57	28.82	±11.70
Weight (Kgs)	92.74	±24.45	82.06	±20.18	100.60	±21.12	93.77	±19.17
BMI (Kg/m^2^)	34.78	±9.28	33.53	±9.96	36.07	±6.47	33.94	±5.84
HbA1c (%)	5.50	±0.67	6.45	±1.83	5.91	±0.69	6.33	±1.16
FBG (mg/dL)	93.69	±8.47	99.06	±42.62	82.64	±15.03	78.26	±26.78
Systolic BP (mmHg)	115.48	±17.72	121.68	±15.65	119.05	±17.75	122.50	±16.79
Diastolic BP (mmHG)	76.72	±13.44	81.36	±11.25	83.48	±13.42	81.86	±9.53
LDL (mg/dL)	95.52	±22.61	110.32	±33.39	94.28	±33.14	70.52	±28.25
HDL (mg/dL)	36.81	±10.60	41.36	±12.76	34.81	±10.05	34.81	±12.88
Triglyceride (mg/dL)	101.29	±33.00	116.63	±79.37	113.50	±91.97	71.98	±29.91
Cholesterol (mg/dL)	160.86	±33.04	158.15	±33.19	153.24	±47.38	121.51	±37.95
Waist Circ (cm)	98.61	±17.64	96.56	±13.77	105.46	±14.93	102.08	±11.92
WHR	0.97	±0.06	0.96	±0.06	1.02	±0.06	0.99	±0.07
Fat Mass (kg)	40.96	±17.26	32.23	±14.55	44.65	±13.01	38.30	±14.65
Fat-Free Mass (kg)	49.65	±9.38	49.44	±13.01	55.96	±12.09	56.78	±12.20
Visceral Fat Area (cm^2^)	137.41	±33.66	111.98	±46.43	154.72	±30.13	139.83	±36.84
Body Fat Percentage	43.21	±9.24	19.62	±22.27	44.04	±7.24	40.52	±10.32
Skeletal Muscle Mass (kg)	28.82	±7.31	27.58	±7.56	31.34	±7.35	31.24	±6.99

**Table 2:** Effect of the spice interventions on cardiometabolic parameters.

Variable	Group	Baseline		Week 6		Week 12		ΔW0–W12	*p*-Value
Systolic BP	Blackseed	115.483	(2.974)	114.655	(2.974)	112.864	(3.017)	−2.619	0.434
	Cinnamon	121.68	(3.203)	115.8	(3.203)	114.64	(3.203)	−7.04	
	Ginger	119.048	(3.495)	115.857	(3.495)	116.857	(3.495)	−2.191	
	Placebo	122.5	(3.414)	120.045	(3.414)	122.545	(3.414)	0.045	
Diastolic BP	Blackseed	76.724	(2.215)	75.828	(2.215)	77.571	(2.266)	0.847	0.299
	Cinnamon	81.36	(2.386)	79.72	(2.386)	77.8	(2.386)	−3.56	
	Ginger	83.476	(2.603)	78.381	(2.603)	79.381	(2.603)	−4.095	
	Placebo	81.864	(2.543)	75.636	(2.543)	80.727	(2.543)	−1.137	
Total	Blackseed	160.859	(7.392)	153.787	(7.392)	147.804	(7.392)	−13.055	0.031
Cholesterol	Cinnamon	158.147	(7.962)	148.248	(7.962)	157.839	(7.962)	−0.308	
	Ginger	153.233	(8.687)	159.063	(8.687)	141.743	(8.687)	−11.49	
	Placebo	121.508	(8.487)	145.13	(8.487)	129.371	(8.487)	7.863	
HDL	Blackseed	36.811	(2.408)	36.811	(2.408)	41.033	(2.408)	4.222	0.2
	Cinnamon	41.363	(2.593)	41.364	(2.593)	49.123	(2.593)	7.76	
	Ginger	34.807	(2.83)	34.808	(2.83)	35.424	(2.83)	0.617	
	Placebo	34.805	(2.764)	34.945	(2.794)	36.513	(2.764)	1.708	
LDL	Blackseed	95.524	(5.284)	92.123	(5.284)	93.382	(5.284)	−2.142	<0.001
	Cinnamon	110.32	(5.691)	96.251	(5.691)	113.741	(5.691)	3.421	
	Ginger	94.282	(6.21)	98.299	(6.21)	88.968	(6.21)	−5.314	
	Placebo	70.515	(6.067)	86.663	(6.067)	76.921	(6.067)	6.406	
Triglycerides	Blackseed	101.288	(11.1)	93.579	(11.1)	93.204	(11.1)	−8.084	0.137
	Cinnamon	116.635	(11.955)	112.715	(11.955)	112.318	(11.955)	−4.317	
	Ginger	113.498	(13.044)	108.89	(13.044)	95.871	(13.044)	−17.627	
	Placebo	71.98	(12.744)	91.082	(12.744)	82.625	(12.744)	10.645	
Fasting	Blackseed	93.694	(4.946)	90.722	(4.946)	86.268	(4.946)	−7.426	<0.001
Blood	Cinnamon	99.057	(5.328)	96.206	(5.328)	97.787	(5.328)	−1.27	
Glucose	Ginger	82.635	(5.813)	86.004	(5.813)	79.232	(5.813)	−3.403	
	Placebo	78.258	(5.679)	94.028	(5.679)	86.817	(5.679)	8.559	
HbA1c	Blackseed	5.5	(0.215)	5.576	(0.215)	5.492	(0.216)	−0.008	<0.001
	Cinnamon	6.448	(0.231)	7.096	(0.231)	5.852	(0.231)	−0.596	
	Ginger	5.91	(0.252)	5.729	(0.252)	5.619	(0.252)	−0.291	
	Placebo	6.327	(0.246)	6.35	(0.246)	6.368	(0.246)	0.041	

**Table 3:** Effects of the spice interventions on anthropometric indices.

Variable	Group	Baseline		Week 6		Week 12		ΔW0–W12	*p*-Value
Weight	Blackseed	92.741	(3.986)	93.134	(3.986)	93.597	(3.986)	0.856	0.278
	Cinnamon	82.056	(4.293)	78.516	(4.3)	81.684	(4.293)	−0.372	
	Ginger	99.272	(4.693)	99.41	(4.684)	99.586	(4.684)	0.314	
	Placebo	93.773	(4.576)	95.507	(4.576)	95.818	(4.576)	2.045	
BMI	Blackseed	34.776	(1.562)	34.938	(1.562)	35.046	(1.562)	0.27	0.221
	Cinnamon	33.532	(1.683)	32.427	(1.688)	34.972	(1.683)	1.44	
	Ginger	35.681	(1.843)	35.652	(1.836)	35.862	(1.836)	0.181	
	Placebo	33.941	(1.794)	34.605	(1.794)	34.191	(1.794)	0.25	
Waist	Blackseed	98.61	(3.085)	95.839	(3.085)	88.046	(3.085)	−10.564	0.051
Circumference	Cinnamon	96.559	(3.322)	90.353	(3.322)	89.427	(3.322)	−7.132	
	Ginger	105.46	(3.625)	101.316	(3.625)	99.286	(3.625)	−6.174	
	Placebo	102.075	(3.542)	101.336	(3.542)	102.364	(3.542)	0.289	
WHR	Blackseed	0.965	(0.012)	0.953	(0.012)	0.965	(0.012)	0	0.073
	Cinnamon	0.956	(0.013)	0.953	(0.013)	0.962	(0.013)	0.006	
	Ginger	1.014	(0.014)	1.005	(0.014)	1.004	(0.014)	−0.01	
	Placebo	0.99	(0.013)	0.98	(0.013)	1.001	(0.013)	0.011	
Fat mass	Blackseed	40.962	(2.818)	40.683	(2.818)	41.338	(2.818)	0.376	0.076
	Cinnamon	32.232	(3.035)	29.904	(3.039)	32.696	(3.035)	0.464	
	Ginger	43.766	(3.316)	43.876	(3.311)	44.138	(3.311)	0.372	
	Placebo	38.295	(3.235)	39.541	(3.235)	38.441	(3.235)	0.146	
Body fat %	Blackseed	43.214	(2.513)	42.697	(2.513)	43.167	(2.523)	−0.047	0.17
	Cinnamon	19.622	(2.706)	18.881	(2.711)	21.886	(2.706)	2.264	
	Ginger	43.662	(2.958)	43.605	(2.953)	43.9	(2.953)	0.238	
	Placebo	40.523	(2.885)	40.818	(2.885)	40.586	(2.885)	0.063	
Visceral	Blackseed	137.41	(6.79)	136.866	(6.79)	136.493	(6.868)	−0.917	0.427
Fat Area	Cinnamon	111.976	(7.313)	113.104	(7.343)	122.324	(7.313)	10.348	
	Ginger	152.706	(8.019)	150.529	(7.979)	153.01	(7.979)	0.304	
	Placebo	139.832	(7.796)	139.309	(7.796)	141.736	(7.796)	1.904	
Fat-Free Mass	Blackseed	49.652	(2.069)	51.283	(2.069)	50.545	(2.069)	0.893	0.815
	Cinnamon	49.444	(2.229)	48.622	(2.237)	48.98	(2.229)	−0.464	
	Ginger	55.516	(2.443)	55.533	(2.432)	55.448	(2.432)	−0.068	
	Placebo	56.783	(2.376)	57.791	(2.376)	57.332	(2.376)	0.549	
Skeletal	Blackseed	28.821	(1.79)	29.29	(1.79)	29.46	(1.855)	0.639	0.442
Muscle Mass	Cinnamon	27.584	(1.928)	26.826	(1.954)	30.972	(1.928)	3.388	
	Ginger	31.908	(2.137)	33.3	(2.104)	31.019	(2.104)	−0.889	
	Placebo	31.236	(2.056)	31.736	(2.056)	31.768	(2.056)	0.532	

WHR: Waist to hip ratio.

## Data Availability

All data informing the results of this study are available upon reasonable request to the corresponding author. The data are not publicly available due to human data privacy regulations at the institute of approval.
