# Correction: Al Dhaheri et al. The Effect of Therapeutic Doses of Culinary Spices in Metabolic Syndrome: A Randomized Controlled Trial. *Nutrients* 2024, *16*, 1685

**DOI:** 10.3390/nu16223791

**Published:** 2024-11-05

**Authors:** Ayesha S. Al Dhaheri, Dana Hasan Alkhatib, Jack Feehan, Leila Cheikh Ismail, Vasso Apostolopoulos, Lily Stojanovska

**Affiliations:** 1 Department of Nutrition & Health Sciences, College of Medicine and Health Sciences, United Arab Emirates University, Al Ain P.O. Box 15551, United Arab Emirates; danakhatib@uaeu.ac.ae (D.H.A.); lily.stojanovska@vu.edu.au (L.S.); 2 Institute for Health and Sport, Victoria University, Melbourne, VIC 8001, Australia; jack.feehan@vu.edu.au (J.F.); vasso.apostolopoulos@vu.edu.au (V.A.); 3 Department of Clinical Nutrition and Dietetics, College of Health Sciences, University of Sharjah, Sharjah 27272, United Arab Emirates; lcheikhismail@sharjah.ac.ae; 4 Nuffield Department of Women’s & Reproductive Health, University of Oxford, Oxford OX1 2JD, UK; 5 Immunology Program, Australian Institute of Musculoskeletal Science (AIMSS), Melbourne, VIC 3021, Australia

In the original publication [1], there was a mistake in Table 1 as published. Incorrect baseline descriptive data were included in the published version of the manuscript. In addition, a correction has been made to Section 3.2, the first three paragraphs. The corrected text appears below:

The clinical and demographic characteristics of the study population are presented as mean ± standard deviation in Table 1. The mean age of the study population was 26.59 ± 8.07, 27.84 ± 12.04, 26.10 ± 9.57, and 28.82 ± 11.70 years old for the black seed, cinnamon, ginger, and placebo groups, respectively, ranging from 19 to 49 years old. The mean body mass index (BMI) of the participants was 34.78 ± 9.28, 33.53 ± 9.96, 36.07 ± 6.47, and 33.94 ± 5.84 kg/m² in the black seed, cinnamon, ginger, and placebo groups, respectively.

All the participants had two or more of the metabolic syndrome risk factors. Waist circumference (WC) was 98.61 ± 17.64, 96.56 ± 13.77, 105.46 ± 14.93, and 102.08 ± 11.92 for the black seed, cinnamon, ginger, and placebo groups, respectively. The systolic blood pressure mean values were 115.48 ± 17.72, 121.68 ± 15.65, 119.05 ± 17.75, and 122.50 ± 16.79 mmHg in the black seed, cinnamon, ginger, and placebo groups, respectively, and 76.72 ± 13.44, 81.36 ± 11.25, 83.48 ± 13.42, and 81.86 ± 9.53 mmHg for the diastolic blood pressure in the black seed, cinnamon, ginger, and placebo groups, respectively. The fasting blood glucose (FBG) mean values were 93.69 ± 8.47, 99.06 ± 42.62, 82.64 ± 15.03, and 78.26 ± 26.78 mg/dL for the black seed, cinnamon, ginger, and placebo groups, respectively. The high-density lipoprotein (HDL) mean values ranged from 34.81 ± 10.05 mg/dL for the ginger group to 41.36 ± 12.76 mg/dL for the cinnamon group, with no significant differences between the ginger, cinnamon, and black seed groups and the placebo group. The triglyceride mean values were the highest in the cinnamon group, 116.63 ± 79.37 mg/dL, and the lowest in the black seed group, 101.29 ± 33.00 mg/dL. No significant differences were found between the ginger, cinnamon, and black seed groups and the placebo group for triglycerides.

The participants demonstrated no significant differences between each treatment group and the placebo group in terms of their age, height, BMI, WC, BP, FBG, HDL, and TG. On the other hand, the weight mean values of the participants ranged from 92.74 ± 25.45 kg for the black seed group to 100.6 ± 21.12 kg for the ginger group. Significant differences were observed in the weight mean values between the placebo group and ginger group and between the placebo group and cinnamon group, as demonstrated in Table 1.

The corrected Table 1 appears below:

The authors state that the scientific conclusions are unaffected. This correction was approved by the Academic Editor. The original publication has also been updated.

## Figures and Tables

**Table 1:** Baseline characteristics of the groups.

Parameter	Black Seed (Mean ± SD)		Cinnamon (Mean ± SD)		Ginger (Mean ± SD)		Placebo (Mean ± SD)	
Age (years)	26.59	±8.07	27.84	±12.04	26.10	±9.57	28.82	±11.70
Weight (Kgs)	92.74	±24.45	82.06	±20.18	100.60	±21.12	93.77	±19.17
BMI (Kg/m^2^)	34.78	±9.28	33.53	±9.96	36.07	±6.47	33.94	±5.84
HbA1c (%)	5.50	±0.67	6.45	±1.83	5.91	±0.69	6.33	±1.16
FBG (mg/dL)	93.69	±8.47	99.06	±42.62	82.64	±15.03	78.26	±26.78
Systolic BP (mmHg)	115.48	±17.72	121.68	±15.65	119.05	±17.75	122.50	±16.79
Diastolic BP (mmHG)	76.72	±13.44	81.36	±11.25	83.48	±13.42	81.86	±9.53
LDL (mg/dL)	95.52	±22.61	110.32	±33.39	94.28	±33.14	70.52	±28.25
HDL (mg/dL)	36.81	±10.60	41.36	±12.76	34.81	±10.05	34.81	±12.88
Triglyceride (mg/dL)	101.29	±33.00	116.63	±79.37	113.50	±91.97	71.98	±29.91
Cholesterol (mg/dL)	160.86	±33.04	158.15	±33.19	153.24	±47.38	121.51	±37.95
Waist Circ (cm)	98.61	±17.64	96.56	±13.77	105.46	±14.93	102.08	±11.92
WHR	0.97	±0.06	0.96	±0.06	1.02	±0.06	0.99	±0.07
Fat Mass (kg)	40.96	±17.26	32.23	±14.55	44.65	±13.01	38.30	±14.65
Fat-Free Mass (kg)	49.65	±9.38	49.44	±13.01	55.96	±12.09	56.78	±12.20
Visceral Fat Area (cm^2^)	137.41	±33.66	111.98	±46.43	154.72	±30.13	139.83	±36.84
Body Fat Percentage	43.21	±9.24	19.62	±22.27	44.04	±7.24	40.52	±10.32
Skeletal Muscle Mass (kg)	28.82	±7.31	27.58	±7.56	31.34	±7.35	31.24	±6.99
